# Neurogenic niches in the brain: help and hindrance of the barrier systems

**DOI:** 10.3389/fnins.2015.00020

**Published:** 2015-02-03

**Authors:** Helen B. Stolp, Zoltán Molnár

**Affiliations:** ^1^Division of Biomedical Engineering and Health Sciences, Department of Perinatal Imaging and Health, King's College LondonLondon, UK; ^2^Department of Physiology, Anatomy and Genetics, University of OxfordOxford, UK

**Keywords:** neurogenesis, neuronal progenitors, neurogenic niche, blood-brain barrier, cerebrovasculature, cerebrospinal fluid, choroid plexus

## Abstract

In the developing central nervous system, most neurogenesis occurs in the ventricular and subventricular proliferative zones. In the adult telencephalon, neurogenesis contracts to the subependyma zone and the dentate gyrus (subgranular zone) of the hippocampus. These restricted niches containing progenitor cells which divide to produce neurons or glia, depending on the intrinsic and environmental cues. Neurogenic niches are characterized by a comparatively high vascular density and, in many cases, interaction with the cerebrospinal fluid (CSF). Both the vasculature and the CSF represent a source of signaling molecules, which can be relatively rapidly modulated by external factors and circulated through the central nervous system. As the brain develops, there is vascular remodeling and a compartmentalization and dynamic modification of the ventricular surface which may be responsible for the change in the proliferative properties. This review will explore the relationship between progenitor cells and the developing vascular and ventricular space. In particular the signaling systems employed to control proliferation, and the consequence of abnormal vascular or ventricular development on growth of the telencephalon. It will also discuss the potential significance of the barriers at the vascular and ventricular junctions in the influence of the proliferative niches.

## Introduction

It is essential that an organ and its blood supply should develop together in synchrony to allow optimal conditions for the different stages of growth, differentiation and changing functional requirements. This co-dependence is particularly evident in the brain, where the vascular network originates from the peri-neural vascular plexus in early embryonic development and undergoes multiple stages of remodeling to meet the changing needs of the complex developing environment (Bautch and James, 2009). Barrier functions of the neurovasculature start from the earliest stages of development and are coupled with other aspects of vascular and neuro development (Ruhrberg and Bautch, 2013).

Close examination of developing brain has led to the hypothesis that signals from the brain parenchyma itself regulate the sites of penetrance by the pioneering vascular branches from the peri-neural plexus, and there is now increasing evidence that variations in normal brain growth can alter the vasculature (Vasudevan et al., 2008) in the same way that altered vascular development affects neural patterning (Javaherian and Kriegstein, 2009). This is, at least partly, due to the presence of a variety of co-signaling systems, e.g., vascular endothelial growth factor (VEGF), Notch, Sonic Hedgehog (Shh) and their receptors, which clearly have a role in both the developing neural system as well as the developing vascular system. The involvement of signaling molecules in the neural and vascular systems, while implying close interaction between vascular and neural development, also makes independent manipulation of the systems difficult, and therefore determination of cause and effect in vascular and neural developmental processes is an intractable issue.

**Figure 1:**
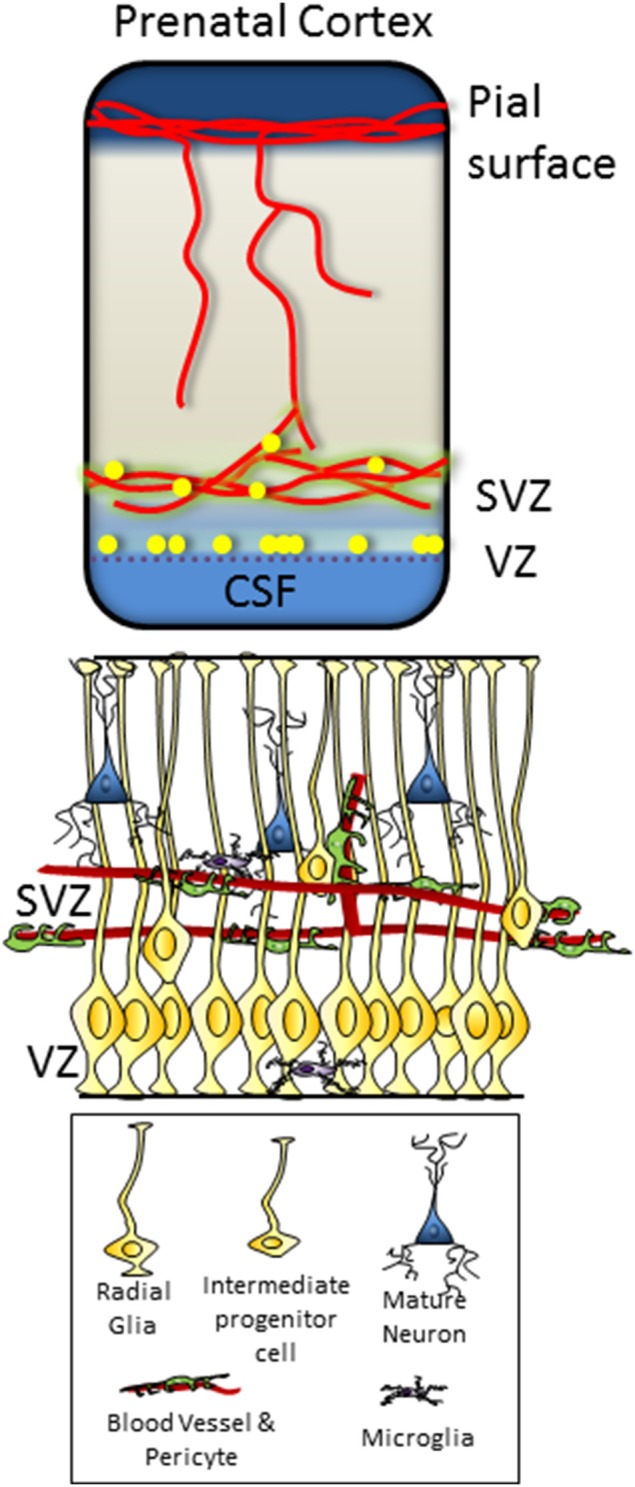
**Dorsal telencephalon in the fetus, showing pial and ventricular vascular plexi and the location of the VZ and SVZ progenitor zones**. Microstructure and cellular component of the VZ and SVZ illustrated below, with VZ progenitors contacting the ventricular surface of the brain and SVZ progenitors in close contact with the vascular plexus.

**Figure 2:**
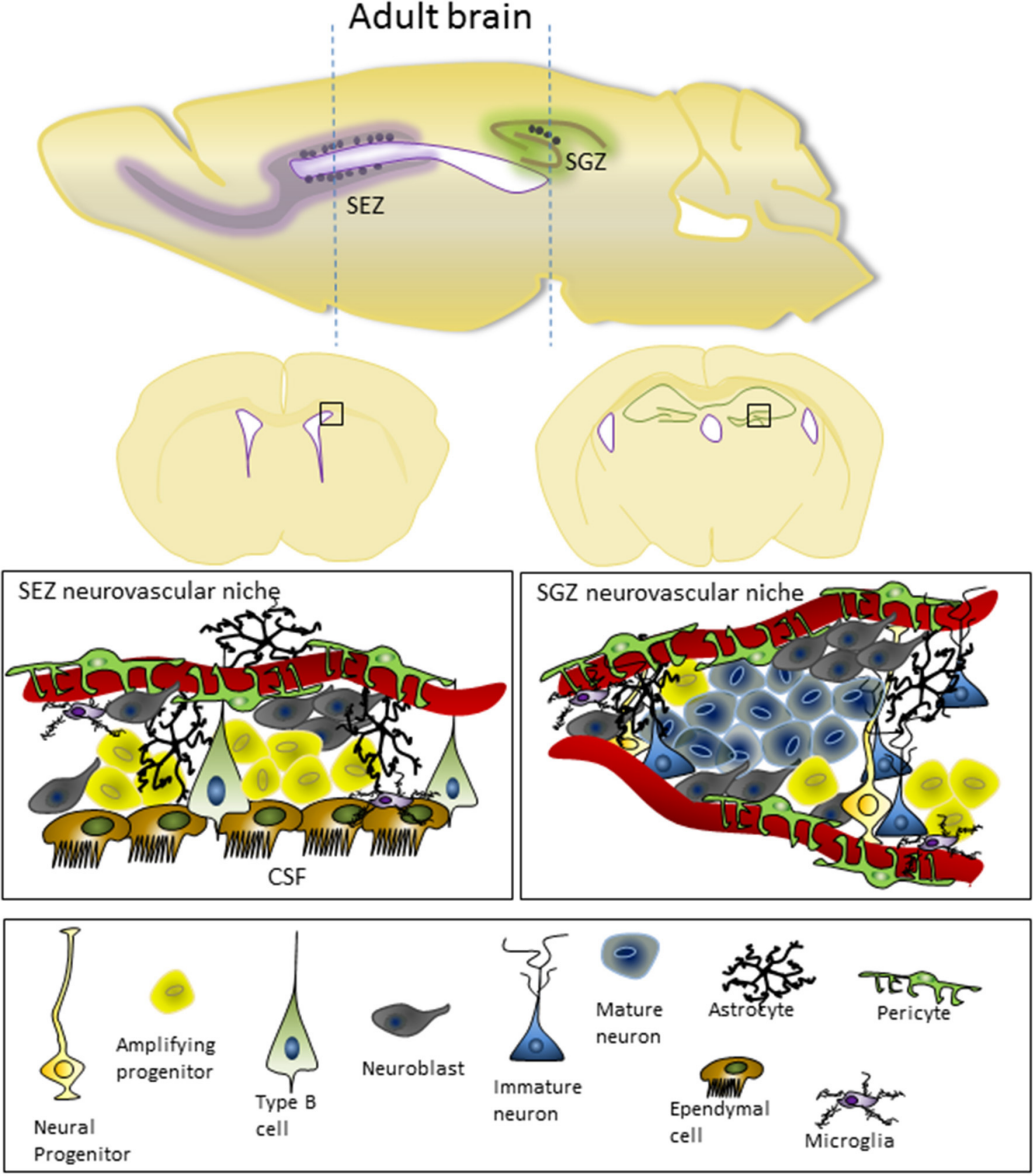
**Adult cortex in the mouse, shown from both sagittal and coronal views, indicating the location of the SEZ and SGZ neurovascular niches**. The microstructure and cellular component of the SEZ and SGZ are shown underneath. In the SEZ, progenitor cells are in close contact with the CSF and ventricular ependymal cells as well as the vasculature, while the SGZ is restricted to influences from the vasculature. For details on the cell types involved and the molecular characteristics of these cells see the recent review from Fuentealba et al. (2012).

During development, the primary regions of proliferation are the ventricular and subventricular zones (VZ and SVZ, respectively, see Figure 1). These are substantive pools of progenitor cells which over time gradually become restricted in their differentiation capacity, and which ultimately produce the diverse neuronal populations of the brain as well as most of the glial cells. In the adult brain (see Figure 2), the proliferative zones are confined to two main areas of the brain, the subependymal zone (SEZ, also called the adult SVZ) and the subgranular zone (SGZ), and the progenitor cells have a more restricted potential fate.

The relationship between neural progenitors and the vasculature has been extensively described, for both the adult SEZ (Shen et al., 2008), and SGZ (Palmer et al., 2000), as well as for the SVZ in the developing cerebral cortex (Stubbs et al., 2009) and developing rostral migratory stream (Nie et al., 2010). In all cases, a clustering of progenitor cells in close proximity with the cerebral endothelial cells was found to occur to at a greater frequency that expected by chance. This observation has led to the hypothesis that the endothelial cells provide a support for the neural progenitors, either structural or chemical, a concept that is now supported by substantial evidence and is discussed in detail below.

The structure and location of the proliferative zones suggest a second regulator of the neurogenic niche, the cerebrospinal fluid (CSF). Both the VZ in the developing brain and the SEZ in the adult have close association with the ventricular surface and the CSF. Therefore, in addition to proliferative support from the cerebrovasculature, trophic regulation/modulation is thought to come from the CSF (Dziegielewska et al., 2000).

The vasculature in the brain is specialized compared to that of the rest of the body. The presence of the blood-brain barrier means there is a tighter control of brain microenvironment as there is limited paracellular transport and reduced vesicular transport of molecules from the blood into the brain. The composition of the CSF is also tightly controlled, in this case by the choroid plexus. The presence of a structural and functional barrier at the epithelial interface of the choroid plexus with the CSF is an essential element of this control. During development, the composition of the CSF is very dynamic, and is considered to be an important for the regulation of a number of neurodevelopmental processed. The specifics of CSF composition regulation and the role of the blood-CSF barrier is beyond the scope of this work, readers are directed to recent reviews by Bueno et al. (2014) and Johansson (2014).

Very few studies have assessed whether the brain barriers actively control progenitor proliferation in neurogenic niches within the brain, but it is likely that at the least the presence of these barriers make an important contribution to the signaling environment of the neurovascular niches. In the case of the VZ, the developmental barrier between the brain and the CSF (Mollgard et al., 1987) may also contribute directly to the proliferative environment.

In this review we will explore the intertwined development of the vasculature and the progenitor pools in the developing brain, the signaling systems common to all neurogenic niches and whether the brain- barrier systems at the endothelial cell and ventricular surface contribute to the control of these specialized brain regions.

## Regulation of vascular growth and barrier formation in the brain

The blood vessel ingrowth from the peri-neural plexus (as shown in Figure 1) to the brain parenchyma is regulated by a number of signaling cues from the parenchyma itself. VEGF signaling is of particular importance, with James et al. (2009) showing that the stereotypical pattern of vascular ingrowth into the neural tube can be disrupted by local over expression of VEGF isotypes (causing ectopic vessel ingrowth) or the VEGF receptor Flt1 (which reduces angiogenesis).

Vasudevan et al. (2008) have shown that the formation of the vascular plexus in the cortex is guided by the neural transcription factors that regulate the proliferation in the pallium and subpallium (e.g., Pax6, Nkx2.1, Dlx1/2). They also put forward the idea that vascular plexi of different origin (e.g., ventral pallium or peri-neural plexus) are differentially controlled by the local proliferative transcription factors. This interplay between neural cells and the vasculature does not stop at proliferative stages of brain growth. The radial migration of vessels from the vascular plexus through the cortex in the human brain appears to be regulated by cxcl12/cxcr4/cxcr7 signaling between the vessels, the radial glial and the perivascular astrocytes (Virgintino et al., 2013). The cxcl12 signaling system appears to be key in regulating many migratory processes in the developing brain, and is probably also involved in controlling progenitor differentiation and migration (discussed below).

Sonic hedgehog (Shh) is a major signaling molecule that regulates both vascular and neural development and may be important in the establishment of the neurovascular niche. Shh has a clear role in regulating neural development, and in the dorsal cortex conditional knockout of Shh or its receptor Smoothened (Smo) results in reduced cell division, and a smaller cortex (Komada et al., 2008). There is also recent evidence that Shh regulates vascular development and blood-brain barrier formation (Alvarez et al., 2011). *In vitro*, Shh in astrocytes conditioned media interacts with receptors on endothelial cells facilitating formation of barrier type properties. Vascular specific knockout of Smo results in impaired blood-brain barrier formation and disrupted extracellular matrix, detectable as early as E14 (Alvarez et al., 2011). It is important to remember that astrocytes are not a major constituent of the brain during very early stages of neurovascular development and therefore more work is required to determine (a) if neural progenitors may be an additional source of Shh, contributing to the control of vascular development in the embryo, and (b) whether vascular Shh contributes a proliferative signal to the neural progenitors.

β-catenin and the Wnt signaling pathway also affect vessel ingrowth from the peri-neural plexus and the structure of the developing vessels. Endothelial specific modifications of β-catenin expression, Wnt7 knockout or delivery of the soluble frizzled-8 receptor to bind extracellular Wnt ligand all cause a disruption in the vascular ingrowth into the parenchyma and malformation of the vessel beds, characterized by enlarged vascular space and thickening of the vascular wall with multiple layers of endothelial cells (Stenman et al., 2008; Daneman et al., 2009). In addition, Wnt signaling has also been shown to affect the barrier properties of the developing parenchymal vessels. Knockout of Wnt7 was associated with reduced GLUT1 expression (Stenman et al., 2008; Daneman et al., 2009), although there was no change in the expression of tight junction proteins. In contrast, endothelial specific β-catenin knockout showed an increased GLUT1 expression (Daneman et al., 2009), indicating that regulation of GLUT and barrier properties by the Wnt pathway is not a clear cut mechanism, and instead is likely to respond to numerous conflicting regulatory signals. However, in general the canonical Wnt signaling has been shown to affect blood-brain barrier permeability and the presence of tight junction proteins at the endothelial cells, where β-catenin gain-of-function stabilizes barrier properties, in response to other changes in Wnt signaling (Liebner et al., 2008). It is possible that the mechanism for this variation is a change in the polarity of the endothelial cells, mediated by Wnt signaling (Artus et al., 2014), which inhibits the formation of tight junctions. The polarity of progenitor cells in the VZ is of clear importance for the formation of junctions and regulating neurogenesis (discussed in more detail below), which provides support for a similar mechanism in the vasculature.

Contact-dependent signaling is another local environmental regulator of growth. McCarty et al. (2002) have shown that deletion of av-integrin causes abnormal vascular ingrowth and alignment into the ganglionic eminence from as early as E10.5. These ultimately led to enlarged vascular space, thinning of the endothelial lining and hemorrhage, and are associated with disruption of the neuroepithelial cells in the surrounding tissue. These disruptions appear to be due to the lack of adhesion with the perivascular basement membrane, rather than endothelial specific signaling or pericyte localization with the vasculature (McCarty et al., 2002), and highlights the importance of all components of the brain microenvironment for the establishment of the neurovascular and neurogenic niche.

## Vascular control of neural proliferation

In turn, vascular development can control proliferation and brain development. For example, ectopic vascular development, induced by *in utero* injection of VEGF into the dorsal cortex, causes a change in distribution of the Tbr2 positive SVZ progenitor cells in conjunction with the new vascular network, and a disruption of radial fibers and axonal ingrowth into the surrounding tissue (Javaherian and Kriegstein, 2009). One of the first studies confirming that cerebral endothelial cells produce neurotophic factors was performed by Leventhal et al. (1999), where co-culture of cells from the adult SEZ with endothelial cells was found to enhance cell survival *in vitro*. These studies confirmed that BDNF produced by the endothelial cells was in fact secreted to affect the neurogenic precursors. Further co-culture studies confirmed that secretions from endothelial cells increased proliferation of precursors and ultimately facilitated the production of a larger number of neurons, of all neuronal classes (Shen et al., 2004). Early work from Tavazoie et al. (2008) suggests that elements of the blood-brain barrier may be different in the SEZ neurovascular niche. They specifically showed that progenitor cells contact blood vessels in place of some of the normal astrocytic endfeet contacts and that this appears to correlate with patches of reduced presence of tight junction proteins and increased permeability. Thus, they hypothesize that cells within the SEZ have an altered exposure to secreted proteins due to changed blood-brain barrier function.

There are certainly many studies showing both secreted protein from endothelial cells, and progenitor-endothelial cell contact is important for maintain proliferation in the SEZ. Transplant studies of neural precursor cells into the SEZ have shown that the chemokine cxcl12, which is produced by endothelial cells, is important for the localization of these cells to the vasculature. Cxcl12 appears to act through the activation of integrin that allows cell binding to the endothelial cell wall, and support migration of differentiating cells out of the SEZ (Kokovay et al., 2010).

Vascular production of VEGF is likely to be due to HIF activation in response to the low oxygen environment of the developing brain. HIF knockout in neural crest cells leads to reduced vascular density in the developing brain, and gross abnormalities in cellular migration (Tomita et al., 2003). There is also substantial hydrocephaly in these animals, possibly suggesting altered neural proliferation and/or the over production of CSF. Tamoxifen-induced knockout of HIF-1α in neural stem cells in the SEZ confirms a constitutive role for this molecule in maintenance of the progenitor population as well as vascular development (Li et al., 2014). As well as altering VEGF production, HIF activation can induce nitric oxide synthesis. Endogenous production of nitric oxide has been shown to negatively regulate progenitor production (Moreno-Lopez et al., 2004), and may act through one of a number of signaling pathways [reviewed by Goldman and Chen (2011)]. Neurotrophin-3 is another molecule released by endothelial cells and found in the CSF that appears to maintain progenitor quiescence/self-renewal through nitric oxide signaling (Delgado et al., 2014). Endothelial-derived pigment epithelium derived factor (PEDF) is another trophic factor that has been show to maintain stem cell renewal in the SEZ, in this case acting through notch signaling (Ramirez-Castillejo et al., 2006; Andreu-Agullo et al., 2009). Local purinergic signaling has also recently be suggested to contribute to self-renewal of progenitors in the SEZ, though it is currently unclear whether these come from the endothelial cells, or other components of the neurogenic niche (see Goldman and Chen, 2011).

Recent work has provided evidence in support of a further neurogenic niche in the adult brain, that of perivascular stem cells that do not proliferate in control conditions, but are upregulated following injury, such as ischemia (Ohira et al., 2010; Nakagomi et al., 2011). The nature of the neurogenic niche for this proliferative population is still not clear, though the recovery of the cortex after ischemia has been shown to be improved by cotransplantation of endothelial cells and neural stem cells (Nakagomi et al., 2009). However, the cells produced from the perivascular progenitors activated by ischemia appear to be more gliogenic than neurogenic (Nakagomi et al., 2011), and may, therefore, have a different relationship with the vasculature. As an extension to this, it has recently been suggested that signaling from the meninges call also regulate proliferative/differentiation decisions made in the VZ and SVZ of the developing brain [discussed by Sockanathan and Gaiano (2009)].

## Role of cell-to-cell contacts in regulation of proliferation

In addition to secreted signaling to neural precursors, the endothelial cells may also support the progenitor population in a contact-dependent manner (contact between type B and endothelial cells in the SEZ is shown in Figure 2). Ephrin and notch signaling have both been shown to be activated by contact between type B progenitors and endothelial cells (Ottone et al., 2014). Notch signaling pathways were up-regulated in cells co-cultured with endothelial cells, and Shen et al. (2004) hypothesized that the expansion of the progenitor population in connected sheets co-cultured with endothelial cells might also induce β-catenin signaling, another indication that junctions between progenitor cells may be essential for proliferation. This is certainly the case in the VZ, where the junctions between the neuroepithelial cells at the CSF-brain barrier appear to be essential for normal proliferation. β-catenin and N-cadherin are important components of these junctions, and disruption of β-catenin in the apical endfeet of the projector cells results in reduced proliferation, increased cell death and cortical malformation (Junghans et al., 2005). Hatakeyama et al. (2014) have also clearly shown that disruption of junctions at the ventricular surface affect proliferation. Electroporation with a dominant-negative form of cadherin, which lack the extracellular domain and disrupts the adherens junctions, resulted in a retraction of apical foot process followed by the production of ectopic neurons within the VZ and a clear abolishment of immunoreactivity of the junctional protein ZO-1 in the adjacent area of the ventricular surface. The upshot of this process was an increased differentiation of neurons over production of progenitors within the electroporated region of the brain. Further work from these researchers suggest that in this experimental paradigm canonical β-caterinin/Wnt signaling was not affected, but that Notch signaling was down-regulated (Hatakeyama et al., 2014). In both endothelial cells and neuroepithelial cell progenitors, the production of delta-like ligand from selected cells in the population appear to regulate Notch signaling in the juxtaposed neighboring cells, and this may facilitate selective population changes like initiating vessel sprouting (e.g., Hellstrom et al., 2007) or selective neuronal differentiation. Further work is required to determine whether this sort of interaction between juxtaposed neural stem cells and endothelial cells is part of the signaling system in the neurovascular niche.

While the ventricular zone in the developing brain is characterized by specialized junctions between the neuroepithelial cells, there are no such junctions between the ependymal cells that make the border with the CSF in the adult SEZ. The absence of these junctions between the ependymal cells appears to be very important for the structure of the SEZ, as progenitor cells are intercalated between the ependymal cells (Shen et al., 2008) and the ependymal cells of the ventricular wall form a rosette like arrangement where projections from the progenitor cells protrude through the center of the rosette to make direct contact with the CSF (Mirzadeh et al., 2008). However, it has been hypothesized that while traditional junctions don't exist between ependymal cells, the maintenance of apical adhesions between cells at the ventricular surface is essential for the formation of this specialized proliferative zone (Mirzadeh et al., 2008). The finding that there is close association of the cell adhesion molecule VCAM1 with the type B1 cells at the center of the pinwheels/rosettes, and that rosette formation is disrupted when VCAM is knocked out (Kokovay et al., 2012), supports this hypothesis.

## CSF-neurogenic niche

Both the VZ in the developing brain and the SEZ in the adult brain have clear connections with the CSF (see Figures 1, 2), and it has been shown that the CSF contains many trophic factors important for regulating proliferation in these zones (Johansson et al., 2010; Falcao et al., 2012; Stolp, 2013). Johansson et al. (2013) showed that knockout of the 4th ventricle choroid plexus by selective Otx2 deletion was sufficient to change the composition of the CSF and proliferation in the cortical VZ. These changes were likely, at least in part, to be mediated by Wnt signaling, which was found to be modulated in the CSF of transgenic animals (Johansson et al., 2013). Insulin-like growth factor (Igf) 2 is another molecule that has been shown to be produced by the choroid plexus to circulate in the CSF during early development. It is then able to interact with the Igf receptors on the ventricular surface and regulate proliferation within the VZ in an age dependent manner (Lehtinen et al., 2011).

The possibility that systemic signaling may directly alter the neurogenic niche has been explored by a number of authors, and it is clear that signaling through inflammatory pathways can cause short- and long-term changes in proliferation in the brain (Stolp et al., 2012; Stolp, 2013). In the developing brain, a systemic inflammatory challenge resulted in decreased proliferation in the VZ, which was associated with local changes in β-catenin immunoreactivity and the integrity of the junctions at the ventricular surface (Stolp et al., 2011). Prenatal inflammation produces a long-term reduction in proliferation, as shown in the SGZ by Graciarena et al. (2013), which is equivalent to that produced by an adult systemic inflammatory response. In this case, the SGZ was affected, rather than the SVZ, implying a tissue specific alteration, which may be more related to the vasculature or local microglia than changes in CSF signaling. Chronic inflammation, such as in models of Multiple Sclerosis, has also been found to result in reduced neurogenesis within the brain, though the mechanisms of this have not been fully explored (Pluchino et al., 2008; Tepavcevic et al., 2011). One potential mediator of this response is IL-1β. The IL-1 receptor is found on the type B cell in the SEZ. IL-1 is produced by the choroid plexus cells and released into the CSF (Kokovay et al., 2012), and is upregulated by peripheral inflammation (Marques et al., 2007). Injection of IL-1β into the lateral ventricles increased VCAM-1 expression in the SEZ and reduced the proliferation of type B cells (Kokovay et al., 2012). Cxcl12 is another typically inflammatory mediator that appears to play an important role in the regulation of the SEZ. As well as being produced by endothelia cells (as described above), it is highly expressed by the ependymal cells of the SEZ, and helps to maintain progenitors in their proliferative state (Kokovay et al., 2010). The contribution of cxcl12 to the neurogenic niches is a clear example of the dual influence of the CSF/ependymal and the microvasculature in neurogenesis, and the stage specific mobilization of progenitors through to differentiated neurons or glia which is essential in the brain [discussed by Kokovay et al. (2010); reviewed by Goldman and Chen (2011)].

## Conclusion

Both the CSF and the vasculature of the brain provide regulatory niches for neurogenesis in the developing and adult brain. Variation in angiogenesis and CSF production clearly affect proliferation in the neurogenic niches as a result of cross-talk between these regions, which appears to be dependent both on secreted tropic factors and contact-dependent signaling pathways. The brain barriers, which regulate the internal environment and contribute to the contact between cells in the neurogenic niches are part of this complex system that can be altered both in systemic and central injury. The localization of neurogenic niches makes them sensitive to circulating soluble factors. Recent work from Villeda et al. (2011) highlights this point, showing that the chemokine CCL11 in the plasma and CSF during aging can regulate neurogenesis in the SGZ, which in turn affects learning and memory. The local and systemic control of the neurogenic niches therefore has significant impact on brain function throughout life. Disruption of neurovascular/CSF signaling in early development is likely to contribute to long-term disruption of neural organization and may be a contributor to neurodevelopmental disorders such as autism, schizophrenia and epilepsy. As such, it is essential that we increase our understanding of the control of neurogenic niches and how local and systemic injury/inflammation affect proliferative and differentiation cues in these regions.

### Conflict of interest statement

The authors declare that the research was conducted in the absence of any commercial or financial relationships that could be construed as a potential conflict of interest.

## Abbreviations

CSF: cerebrospinal fluid
SEZ: subependymal zone/also called the adult SVZ
SGZ: subgranular zone
Shh: sonic hedgehog
Smo: smoothened receptor
SVZ: subventricular zone
VCAM: vascular cell adhesion molecule
VEGF: vascular endothelial growth factor
VZ: ventricular zone.
